# Non-Response to Antibiotic Treatment in Adolescents for Four Common Infections in UK Primary Care 1991–2012: A Retrospective, Longitudinal Study

**DOI:** 10.3390/antibiotics5030025

**Published:** 2016-07-04

**Authors:** Ellen Berni, Laura A. Scott, Sara Jenkins-Jones, Hanka De Voogd, Monica S. Rocha, Chris C. Butler, Christopher Ll. Morgan, Craig J. Currie

**Affiliations:** 1 Global Epidemiology and Medical Statistics, Pharmatelligence, Cardiff CF14 3QX, UK; ellen.berni@pharmatelligence.co.uk (E.B.); laura.scott@pharmatelligence.co.uk (L.A.S.); sara.jenkins-jones@pharmatelligence.co.uk (S.J.-J.); chris.morgan@pharmatelligence.co.uk (C.Ll.M.); 2 Mylan Established Pharmaceuticals Division, Weesp 1381 CP, The Netherlands; hanka.devoogd@mylan.com (H.V.); monica.esrocha@gmail.com (M.S.R.); 3 Nuffield Department of Primary Care Health Sciences, University of Oxford, Oxford OX2 6GG, UK; christopher.butler@phc.ox.ac.uk; 4 Cochrane Institute of Primary Care and Public Health, Cardiff University, The Pharma Research Centre, Abton House, Wedal Road, Cardiff CF14 3QX, UK

**Keywords:** otitis media, adolescents, primary care, first line, respiratory tract infection, skin infection, treatment response, treatment failure, antibiotics

## Abstract

We studied non-response rates to antibiotics in the under-reported subgroup of adolescents aged 12 to 17 years old, using standardised criteria representing antibiotic treatment failure. Routine, primary care data from the UK Clinical Practice Research Datalink (CPRD) were used. Annual, non-response rates by antibiotics and by indication were determined. We identified 824,651 monotherapies in 415,468 adolescents: 368,900 (45%) episodes for upper respiratory tract infections (URTIs), 89,558 (11%) for lower respiratory tract infections (LRTIs), 286,969 (35%) for skin/soft tissue infections (SSTIs) and 79,224 (10%) for acute otitis media (AOM). The most frequently prescribed antibiotics were amoxicillin (27%), penicillin-V (24%), erythromycin (11%), flucloxacillin (11%) and oxytetracycline (6%). In 1991, the overall non-response rate was 9.3%: 11.9% for LRTIs, 9.5% for URTIs, 7.1% for SSTIs, 9.7% for AOM. In 2012, the overall non-response rate was 9.2%. Highest non-response rates were for AOM in 1991–1999 and for LRTIs in 2000–2012. Physicians generally prescribed antibiotics to adolescents according to recommendations. Evidence of antibiotic non-response was less common among adolescents during this 22-year study period compared with an all-age population, where the overall non-response rate was 12%.

## 1. Introduction

Microbial resistance in adults and children is increasing worldwide, posing a major threat to public health [1]. Numerous strains of bacteria are becoming more difficult and time-consuming to treat [2], and now some infections cannot be treated with antibiotics [3]. Nevertheless, general practitioners (GPs) are thought to rarely report antibiotic resistance and believe that the problem is out of their control [4].

In order to address non-response to antibiotic treatment, it is essential that patterns of non-response are understood. Microbial resistance typically affects hospitalised patients, more so than in primary care settings, although antibiotics are routinely prescribed for respiratory tract infections in both primary and secondary care [5]. Considering the wide range of antibiotics available, and the different types of microbial infections, the impact of antibiotic resistance has proven difficult to characterise [6]. Extensive prescribing of antibiotics is considered to be the cause of high levels of antibiotic resistance and corresponding treatment non-response [7]. In adolescents, there is a high possibility of improper and over-prescribing of antibiotics, particularly when the prevalence of respiratory tract infections and acute otitis media (AOM) is high [8]. Children with acute respiratory tract infections treated with penicillin had reported resistance levels to be as high as 52% [9]. Nevertheless, data characterising antibiotic resistance in this younger age group are limited.

Concern regarding the effectiveness of antibiotics in adolescents has emerged. During clinical development, this age group is often studied less thoroughly than children aged 12 years and younger. Failure to respond to antibiotics does not necessarily imply a causal association with antibiotic resistance [10]. Many patients who are prescribed antibiotics in primary care are unlikely to benefit because they are suffering from viral infections. Nevertheless, information about non-response trends is important, as this may help clinicians share information with their patients about potential harm and benefits. 

In our previous study [11] marking the 25th anniversary of the availability of clarithromycin, we reported that antibiotic treatment non-response rates for four common infection classes in primary care increased by 12% from 1991 to 2012. Here, given the emerging concern regarding the effectiveness of antibiotics in adolescents, our existing real-world data set was re-analysed to evaluate antibiotic non-response rates in the adolescent age group and compared these with the original study cohort, which contained patients of all ages.

## 2. Methods

### 2.1. Data Source

Data were extracted from the Clinical Practice Research Datalink (CPRD), a longitudinal anonymised research database drawn from nearly 700 primary care practices in the UK. This database contains demographic, diagnostic, treatment and referral information for approximately 8% of the UK population [12].

The data used in this study was a subgroup of the dataset from our recent study [11] and consisted of patients aged from 12 to 17 years that received prescriptions for antibiotics. 

The current study received ethical approval from CPRD’s Independent Scientific Advisory Committee on 31 October 2013, protocol number 13_168R.

### 2.2. Classification of Infection

**Figure 1:**
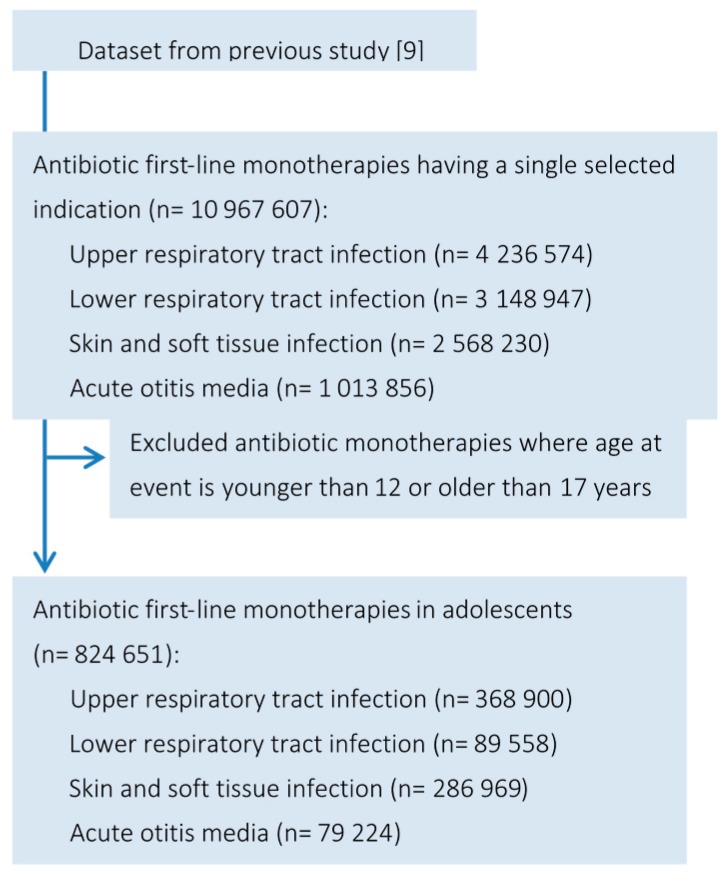
Data flow diagram for selection of antibiotic monotherapies in adolescents reported in the Clinical Practice Research Datalink.

Prescriptions for antibiotics were selected for four relevant infection classes: upper respiratory tract infections (URTIs), which included tonsillitis, laryngitis, pharyngitis and sinusitis; lower respiratory tract infections (LRTIs), including pneumonia, whooping cough and bronchitis; skin and soft tissue infections (SSTIs), including acne, abscesses, impetigo and cellulitis; and acute otitis media (AOM). These categories were the same as in our previous study [11]. The index date was set as the date of initiation of antibiotic monotherapy. Figure 1 illustrates selection of study data.

### 2.3. Identification of Antibiotic Treatments

Antibiotic monotherapy episodes were defined here as one or more consecutive prescriptions for a single antibiotic separated by no more than 30 days and uninterrupted by prescriptions for other antibiotic drug substances. Only first-line monotherapies, i.e. where there were no prescriptions for a different antibiotic in the 30 days prior to initiation, were included in this study. Monotherapy episodes were excluded if they began before 1991 or after 2012, or if the interval from the later of the patient’s registration date and the practice’s up-to-standard date to initiation was less than 365 days.

### 2.4. Infection Rates and Antibiotic Prescription over Time

In a separate procedure (not illustrated), we measured background infection rates over time for each infection class as the proportion of all GP consultations in which the GP recorded a diagnosis of that infection in adolescents. Additionally, we determined the proportion of these infection-related consultations in which an antibiotic was prescribed. The prescription pattern of the five most commonly prescribed antibiotics over the research period in each infection is presented. 

### 2.5. Antibiotic Treatment Non-Response Rates

For each year of the studied period, we evaluated the proportion of antibiotic monotherapies that resulted in treatment non-response. We defined antibiotic treatment non-response as the earliest occurrence of any of five events: (1) prescription of a different antibiotic within 30 days of the initial antibiotic prescription; or (2) GP record of admission to hospital with a diagnosis of infection within 30 days of initial antibiotic prescription; or (3) GP referral to an infection-related specialist service within 30 days of initial antibiotic prescription; or (4) a GP record of emergency department visit within three days of antibiotic initiation; or (5) a GP record of death with an infection-related clinical code, within 30 days of initial antibiotic prescription.

Treatment non-response rates are presented overall and for the five most frequently prescribed antibiotics within each infection class. Over the 22 years observed, the number of patients and participating practices increased, requiring treatment non-response rates to be averaged over the first five-year period (1991–1995) in order to create a more stable baseline for comparison with those in the last five-year period (2008–2012).

### 2.6. Statistical Analysis 

Treatment non-response rates were presented as standardised ratios of observed rates to predicted rates for each year, using 1991 as the index year. Standardised ratios were adopted because of variations in antibiotic prescription patterns and age dispersion. Indexed treatment non-response rates were calculated using a matrix of infection subclass, antibiotic drug and gender; rates from 1991 were used to predict expected number of treatment non-responses, and, subsequently, to calculate the ratio.

## 3. Results

In this study 824,651 antibiotic monotherapies were prescribed to 415,468 adolescents. Of these, 368,900 (45%) therapies were for URTIs; 89,558 (11%) for LRTIs; 286,969 (35%) for SSTIs; and 79,224 (10%) for AOM (Figure 1). 

### 3.1. Baseline Characteristics

The mean age of the adolescents in 1991–1995 was 14.5 years (Standard deviation (SD) 1.7 years) and in 2008–2012, 14.8 years (SD 1.7 years) (Table 1). Those with a diagnosis of AOM were younger (1991–1995: 13.9, SD 1.6 years and 2008–2012: 14.1, SD 1.7 years) and those with SSTIs slightly older (1991–1995: 15.1, SD 1.5 years and 2008–2012: 15.1, SD 1.5 years). Gender distribution was generally comparable across infection classes (1991–1995: 49.9% female; 2008–2012: 53.0% female), although URTIs were more common in females (1991–1995: 54.1%; 2008–2012: 58.7%) and LRTIs more common in males (1991–1995: 53.8%; 2008–2012: 53.1%).

Within the LRTI infection class, the proportion of co-medications increased from 1991–1995 to 2008–2012 from 2.8% to 8.0% for systemic corticosteroids, 22.3% to 29.4% for bronchodilators and 9.8% to 16.3% for inhaled corticosteroids. The proportion of co-medications in the other three infection classes remained generally constant from the first to the latter five-year period. The proportion of adolescent smokers was high (20%–27%) in the 1991–1995 period, but decreased to 5%–12% in the period 2008–2012 (Table 1).

### 3.2. Consultation Rates 

**Figure 2:**
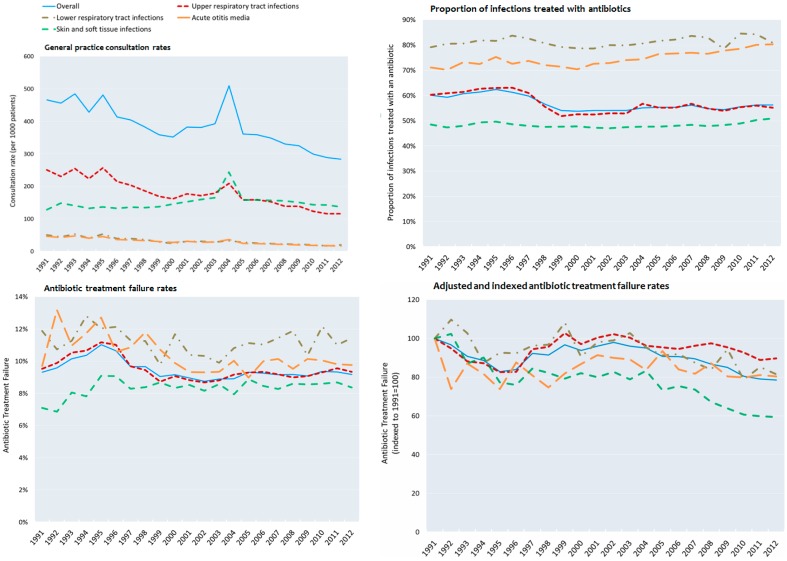
Consultation rates for the selected infection classes, proportion of infections in the selected classes treated with an antibiotic, antibiotic treatment non-response rates by infection class, and adjusted and indexed treatment non-response rates (indexed to 1991 = 100, and adjusted for age, sex, and type of antibiotic treatment used) observed between 1991 and 2012.

GP consultation rates for the four infection classes, with or without antibiotic treatment, decreased over time (Figure 2). The all-cause consultation rate decreased from 466 consultations per 1000 registered patients per year in 1991 to 284 in 2012, a decrease of 39.1% (*p* < 0.01). The change in consultation rates varied by type of infection. Rates of consultations per 1000 patients per year decreased from 251 to 110 (*p* < 0.01) for URTIs, from 46 to 16 (*p* < 0.01) for AOM, and from 50 to 20 (*p* < 0.01) for LRTIs. Consultation rates for SSTI increased from 129 to 137 consultations per 1000 patients per year (*p* < 0.01).

### 3.3. Most Commonly Prescribed Antibiotics in Adolescents

The five most frequently prescribed antibiotics were amoxicillin (27.3%), penicillin-V (24.2%), erythromycin (11.3%), flucloxacillin (10.5%) and oxytetracycline (5.9%). Lymecycline (4.1%), co-amoxiclav (1.5%), doxycycline (1.4%) and clarithromycin (1.3%) were less frequently prescribed; the remaining antibiotics were prescribed in even lower quantities and they are reported together in a combined group termed “Others” (12.5%).

**Figure 3:**
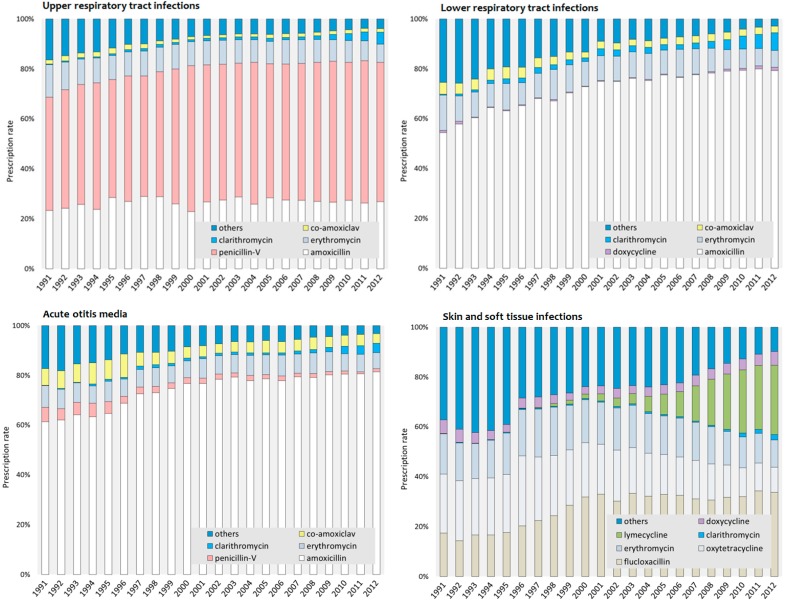
Five most commonly prescribed antibiotics between 1991 and 2012 by selected infection class.

The prescription pattern of the most frequently prescribed antibiotics in each infection class, for each year between 1991 and 2012 is illustrated in Figure 3. For URTIs, penicillin-V (53.6%) was the most commonly prescribed antibiotic over the whole period. Amoxicillin was the most frequently prescribed antibiotic for the treatment of LRTIs (73.3%) and AOM (75.8%). The five most commonly prescribed antibiotics for SSTIs were flucloxacillin (30.4%), oxytetracycline (17.0%), erythromycin (15.3%), lymecycline (11.8%) and doxycycline (4.0%). In this infection class, the combined group of other antibiotics accounted for up to 21% of prescriptions. 

In adolescents, skin and soft tissue infection prescriptions were more prominent than in the original, all-age study (35% vs. 23%), with therapies associated with acne comprising more than half of the antibiotic therapies in the adolescent study [11]. By contrast, antibiotic therapy for lower respiratory infection was relatively rare compared with the overall study (11% vs. 29%). Antibiotic therapies for upper respiratory infections and acute otitis media occurred in proportions comparable with the original study (45% vs. 39%, and 10% vs. 9%, respectively).

A consistent prescription rate over the 22 years was generally observed in the five most frequently prescribed antibiotics for URTIs. However, antibiotics prescribed for SSTIs showed a varying pattern over time (Figure 3). For example, lymecycline showed the greatest increase in therapies since it was introduced in 1996, increasing from 0.1% to 27.9%. The proportion of oxytetracycline therapies showed the greatest decrease: from 23.6% to 10.0%.

The most frequently prescribed antibiotics in each infection class remained the same from the first five-year period to the last five-year period, albeit with changes in their ranking of relative frequency (Table 2). Penicillin-V remained the most prescribed antibiotic for the treatment of URTIs in the first and last five-year periods. For LRTIs and AOM, the most frequently prescribed antibiotic in both five-year periods was amoxicillin. In the first five-year period, oxytetracycline was the second-most-prescribed antibiotic for the treatment of SSTIs in adolescents.

### 3.4. Failure to Respond to Antibiotics in Adolescents

The overall antibiotic non-response rate for the four infection classes was 9.5%, with a non-linear increase from 9.3% in 1991, peaking at 11.0% in 1995, and then declining to 9.2% in 2012. In 1991, the treatment non-response rates were 11.9% for LRTIs, 9.5% for URTIs, 7.1% for SSTIs and 9.7% for AOM. In 2012, treatment non-response rates were 11.4% for LRTIs, 9.4% for URTIs, 8.4% for SSTIs and 9.8% for AOM (Figure 2). SSTIs had the lowest treatment non-response rate in each five-year period (7.8% in 1991–1995 and 8.6% in 2008–2012). The highest treatment non-response rates were for AOM in 1991–1995 (11.7%) and for LRTIs from 2008 to 2012 (11.4%) (Table 2).

**Figure 4:**
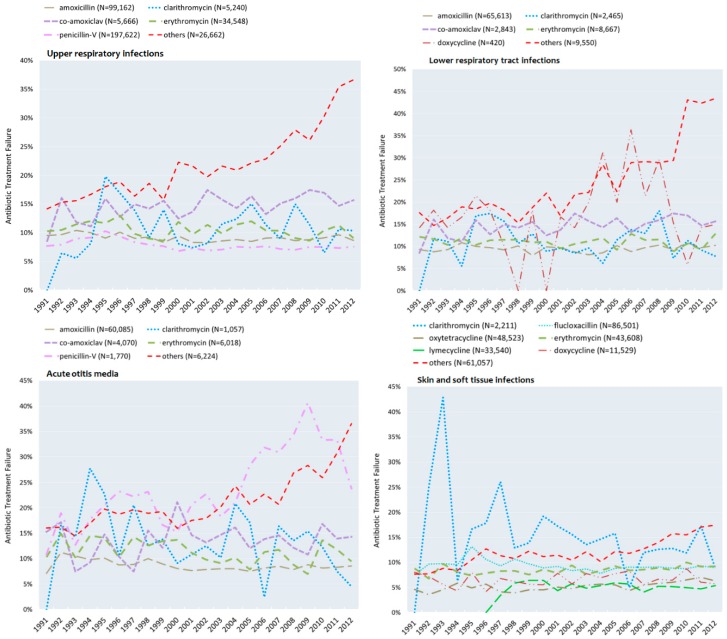
Antibiotic treatment non-response rates observed from 1991 to 2012 for the most commonly prescribed antibiotics in each selected infection class. “Other” antibiotics are defined in Table 2.

The two most frequently prescribed antibiotics for URTIs, penicillin-V and amoxicillin, both had stable non-response rates, ranging from 7% to 11% over the study period (Figure 4). Within the five most commonly prescribed antibiotics for LRTIs, amoxicillin, erythromycin and co-amoxiclav had constant treatment non-response rates of between 5% and 17% over the period (Figure 4). The five most frequently prescribed antibiotics for SSTIs had treatment non-response rates of between 4% and 13%, except for the antibiotic lymecycline, which only became available in 1996 (Figure 4).

For AOM, the treatment non-response rates of amoxicillin, erythromycin and co-amoxiclav remained stable over time (Figure 4). In contrast, for penicillin-V, treatment non-response rates increased markedly: from 16% in 1991–1995 to 33% in 2008–2012, with a rate of 40% in 2009 (Figure 4, Table 2).

### 3.5. Adjusted Rates of Non-Response of Antibiotic Treatment

After adjusting for antibiotic, index year and infection class, the rate of LRTI treatment non-response initially increased by 9% from 1991 to 1992 and then decreased from 1992 to 2012 by 28.3%. For SSTIs, URTIs and AOM, rates of antibiotic treatment non-response decreased from 1991 to 2012 by 40.5%, 10.2% and 19.6%, respectively. The overall adjusted treatment non-response rate decreased by 21.5% over the study period (Figure 2).

## 4. Discussion

This study examined non-response, in adolescents, to antibiotics prescribed for four common infection classes. We found that primary care physicians prescribe antibiotics to adolescents as they do for adults, rarely prescribing non-recommended compounds. In general, response to antibiotic treatment in adolescents was better than that in the all-age population.

We selected data for patients aged from 12 to 17 years from the dataset created for our original CPRD study [11] and analysed their demographic changes, consultation patterns, antibiotic treatment patterns, and treatment response rates. The proportion of adolescents in the original dataset was 7.1%, which is roughly comparable with the relative number of adolescents in the UK population over the research period [13], and the proportion of therapies prescribed to adolescents was 7.5%.

The most frequently prescribed antibiotics for upper respiratory tract infections in adolescents—penicillin-V, amoxicillin, erythromycin, co-amoxiclav and clarithromycin—were also frequent choices in the original cohort, and these are also listed in the guidelines as primary choices for bacterial infections [14]. However, antibiotics such as cefalexin, oxytetracycline, trimethoprim, cefaclor and doxycycline, which were in the top 10 for upper respiratory tract infections in the original cohort [11], were hardly prescribed to adolescents. The treatment non-response rates for penicillin and amoxicillin were below 10% in the first five years of the study period (1991–1995) and, on average, over the last five years (2008–2012). Non-response rates for the macrolides erythromycin and clarithromycin were around 10%. Co-amoxiclav and the combined “Other” antibiotics had higher and increasing treatment non-response rates. When prescribed for URTIs, penicillin and amoxicillin had lower treatment non-response rates in adolescents, compared with the original cohort.

Lower respiratory tract infections were uncommon in adolescents (only 2.6% of the infections). The antibiotics most frequently prescribed for lower respiratory tract infections: amoxicillin, erythromycin, co-amoxiclav, clarithromycin and doxycycline, were also the most frequently prescribed in the original cohort. Non-response rates for amoxicillin and the macrolides erythromycin and clarithromycin in adolescents were stable and at or below 10% throughout the study period, whilst in the original cohort, the respective non-response rates were between 15% and 20%, probably reflecting the large proportion of older and frailer patients.

During the first five years of the study period, many different antibiotics were prescribed for the treatment of skin infections in adolescence. Over time and with the introduction of lymecycline in the UK in 1996, the pattern of prescription for this infection class became more defined, with flucloxacillin and lymecycline together being prescribed in more than half of the cases.

In acute otitis media in adolescents, the top five antibiotics were amoxicillin, erythromycin, co-amoxiclav, penicillin-V and clarithromycin. In these adolescents as well as in the original study cohort, the majority of cases of AOM in the last five years of the study were treated with amoxicillin (80%). Amoxicillin, erythromycin and clarithromycin had the lowest average treatment non-response rates, between 8% and 11%, over the last five years of the study; these rates were lower than those reported for the original cohort. 

Most respiratory tract infections managed in UK primary care are viral in origin, therefore a large proportion of the infections included in our study would not have responded to antibiotic treatment. Surveillance linked to clinical findings and outcomes was not carried out on a systematic basis during this time period in the UK. We do not therefore know whether changing pathogens and antibiotic sensitivities could have accounted for in the trends described. Point-of-care tests are not used in UK primary care for respiratory tract infections. The numbers consulting with coughs and colds as well as the proportion of coughs and colds treated with antibiotics in primary care increased between 2004 and 2011 [15].

## 5. Limitations

The adolescents formed a small subgroup of the original, all-age population and they were not excluded from the overall population values reported here. Had we compared the adolescent subgroup with the original population minus adolescent patients, the comparisons might have been more distinct. Differences observed here may therefore be somewhat underestimated but are still valid. 

For infections requiring long-term antibiotic use, our algorithm may be overestimating rates of non-response because the prescription of different antibiotics in ongoing therapy would have been taken to signify non-response. Similarly, some infection episodes may have been misclassified as non-responsive because the initial therapy was followed within 30 days by the prescription of another antibiotic for a different indication.

## 6. Conclusions

Adolescents prescribed antibiotics are usually clinically managed as adults, and research on antibiotic non-response in this age group is scarce. In this study, we evaluated antibiotic prescription patterns and non-response rates for the treatment of four common infection classes in adolescents. Our data suggest that primary care physicians prescribe antibiotics to adolescents in line with current guidelines regarding initial prescriptions and, using our definition of antibiotic non-response, that adolescents respond generally better to these antibiotics compared with the general population as a whole. Non-response to antibiotics does not imply a causal link with antibiotic resistance, as many of the infections for which antibiotics are prescribed are viral. However, antibiotic resistance may be relevant in a minority of infections, and knowledge of non-response rates may be useful to inform discussion with patients about potential risks and benefits of antibiotic treatment for common infections. 

## Figures and Tables

**Table 1:** Baseline characteristics by infection class and by early and late time period.

Baseline Characteristic	Upper Respiratory		Lower Respiratory		Skin and Soft Tissue		Acute Otitis Media		Overall	
	1991–1995	2008–2012	1991–1995	2008–2012	1991–1995	2008–2012	1991–1995	2008–2012	1991–1995	2008–2012
No of patients, *n*	32,150	73,528	10,063	20,259	13,768	66,937	8614	18,179	50,157	151,200
Antibiotic monotherapies, *n*	52,830	99,726	13,258	23,529	22,566	100,719	10,917	20,714	99,571	244,688
Male, *n* (%)	14,745 (45.9%)	30,354 (41.3%)	5411 (53.8%)	10,754 (53.1%)	7635 (55.5%)	33,426 (49.9%)	4309 (50.0%)	8510 (46.8%)	25,151 (50.1%)	71,113 (47.0%)
Female, *n* (%)	17,405 (54.1%)	43,174 (58.7%)	4652 (46.2%)	9505 (46.9%)	6133 (44.5%)	33,511 (50.1%)	4305 (50.0%)	9669 (53.2%)	25,006 (49.9%)	80,087 (53.0%)
Age, years, mean (SD)	14.4 (1.7)	14.7 (1.7)	14.4 (1.7)	14.6 (1.7)	15.1 (1.5)	15.1 (1.5)	13.9 (1.6)	14.1 (1.7)	14.5 (1.7)	14.8 (1.7)
Smoking, *n* (%):										
Never or not known	24,285 (46.0%)	63,936 (64.1%)	6032 (45.5%)	15,446 (65.6%)	11,216 (49.7%)	71,795 (71.3%)	4841 (44.3%)	12,127 (58.5%)	46,374 (46.6%)	163,304 (66.7%)
Ex–smoker	1575 (3.0%)	1085 (1.1%)	467 (3.5%)	277 (1.2%)	634 (2.8%)	863 (0.9%)	318 (2.9%)	120 (0.6%)	2994 (3.0%)	2345 (0.9%)
Current	12,918 (24.5%)	8646 (8.7%)	3520 (26.6%)	2645 (11.2%)	4481 (19.9%)	5044 (5.0%)	2445 (22.4%)	1085 (5.2%)	23,364 (23.5%)	17,420 (7.1%)
BMI, kg·m^−2^, mean (SD)	21.7 (4.4)	23.0 (5.5)	21.5 (4.6)	22.8 (5.6)	21.6 (3.9)	22.9 (5.2)	21.5 (4.7)	23.3 (6.1)	21.6 (4.4)	22.9 (5.4)
Blood pressure, mmHg, mean (SD):										
Systolic	112.3 (12.3)	111.8 (12.4)	112.2 (13.0)	112.0 (12.7)	113.8 (12.4)	112.6 (12.5)	111.9 (13.6)	111.6 (12.7)	112.6 (12.6)	112.1 (12.5)
Diastolic	69.0 (8.8)	68.1 (8.7)	68.7 (8.6)	68.2 (9.1)	69.6 (8.5)	68.1 (8.6)	68.2 (8.9)	68.1 (9.1)	69.0 (8.7)	68.1 (8.7)
Co–medications, *n* (%):										
Systemic corticosteroid	201 (0.4%)	932 (0.9%)	370 (2.8%)	1881 (8.0%)	63 (0.3%)	348 (0.3%)	40 (0.4%)	97 (0.5%)	674 (0.6%)	3258 (1.3%)
Bronchodilator	3181 (6.0%)	7058 (7.1%)	2,951 (22.3%)	6917 (29.4%)	856 (3.8%)	3963 (3.9%)	551 (5.0%)	1138 (5.5%)	7539 (7.6%)	19,076 (7.8%)
Inhaled corticosteroid	1673 (3.2%)	4444 (4.5%)	1296 (9.8%)	3840 (16.3%)	418 (1.9%)	2560 (2.5%)	334 (3.1%)	758 (3.7%)	3721 (3.7%)	11,602 (4.7%)

*n* = number, SD = standard deviation.

**Table 2:** Average antibiotic therapy and treatment non-response rates (%) by infection class and by early and late time period with rank order in parentheses.

Infection Site	Antibiotic	Therapy Rates				Antibiotic Treatment Non-Response Rates			
		Average 1991–1995		Average 2008–2012		Average 1991–1995		Average 2008–2012	
		% (Rank Order)		% (Rank Order)		% (Rank Order)		% (Rank Order)	
URTIs	Penicillin-V	47.9	(1)	56.0	(1)	8.8%	(2)	7.4%	(1)
	Amoxicillin	25.6	(2)	26.8	(2)	9.7%	(3)	9.0%	(2)
	Others ^a^	13.5	(3)	4.5	(4)	16.0%	(6)	31.3%	(6)
	Erythromycin	10.5	(4)	8.4	(3)	11.2%	(4)	9.7%	(3)
	Co-amoxiclav	2.0	(5)	1.5	(6)	12.7%	(5)	16.2%	(5)
	Clarithromycin	0.5	(6)	2.8	(5)	8.0%	(1)	10.8%	(4)
LRTIs	Amoxicillin	60.9	(1)	79.1	(1)	9.6%	(2)	10.1%	(1)
	Others ^b^	22.3	(2)	4.5	(4)	17.2%	(6)	37.4%	(6)
	Erythromycin	10.6	(3)	7.9	(2)	11.3%	(3)	10.7%	(2)
	Co-amoxiclav	4.5	(4)	2.9	(5)	12.6%	(4)	13.8%	(4)
	Clarithromycin	1.2	(5)	4.8	(3)	9.1%	(1)	10.7%	(3)
	Doxycycline	0.5	(6)	0.9	(6)	17.0%	(5)	16.0%	(5)
SSTIs	Others ^c^	40.4	(1)	13.0	(3)	8.6%	(4)	15.9%	(7)
	Oxytetracycline	23.2	(2)	12.1	(5)	4.7%	(1)	6.3%	(2)
	Flucloxacillin	16.6	(3)	32.4	(1)	9.9%	(5)	9.0%	(4)
	Erythromycin	15.4	(4)	12.8	(4)	8.2%	(3)	9.1%	(5)
	Doxycycline	4.2	(5)	4.6	(6)	6.6%	(2)	6.7%	(3)
	Clarithromycin	0.2	(6)	1.3	(7)	18.2%	(6)	12.7%	(6)
	Lymecycline	-	-	23.8	(2)	-	-	5.1%	(1)
AOM	Amoxicillin	63.4	(1)	80.3	(1)	9.7%	(1)	8.4%	(1)
	Others ^d^	15.6	(2)	4.0	(4)	16.7%	(6)	29.7%	(5)
	Erythromycin	7.9	(3)	7.5	(2)	12.8%	(3)	10.1%	(2)
	Co-amoxiclav	7.6	(4)	4.5	(3)	12.8%	(2)	13.6%	(4)
	Penicillin-V	5.0	(5)	1.2	(6)	16.2%	(4)	33.1%	(6)
	Clarithromycin	0.5	(6)	2.5	(5)	16.3%	(5)	10.7%	(3)

AOM, acute otitis media; LRTIs, lower respiratory infections; SSTIs, soft and skin tissue infections; URTIs, upper respiratory tract infections. ^a^ Others (URTIs): 19 antibiotics, of which the 10 most commonly prescribed were doxycycline, trimethoprim, flucloxacillin, cefalexin, lymecycline, oxytetracycline, azithromycin, cefaclor, nitrofurantoin, minocycline; ^b^ Others (LRTIs): 14 antibiotics (top 10: penicillin-V, cephalexin, azithromycin, trimethoprim, flucloxacillin, oxytetracycline, lymecycline, cefaclor, ciprofloxacin, cefradine); ^c^ Others (SSTIs): 18 antibiotics (top 10: amoxicillin, co-amoxiclav, minocycline, trimethoprim, penicillin-V, tetracycline, co-fluampicil, cephalexin, ciprofloxacin, nitrofurantoin); ^d^ Others (AOM): 15 antibiotics (top 10: flucloxacillin, cefalexin, trimethoprim, ciprofloxacin, doxycycline, lymecycline, oxytetracycline, cefaclor, metronidazole, co-fluampicil).
